# Localization of annexins A1 and A2 in the respiratory tract of healthy calves and those experimentally infected with *Mannheimia haemolytica*

**DOI:** 10.1186/s13567-014-0134-3

**Published:** 2015-02-14

**Authors:** Chandrika Senthilkumaran, Joanne Hewson, Theresa L Ollivett, Dorothee Bienzle, Brandon N Lillie, Mary Ellen Clark, Jeff L Caswell

**Affiliations:** Department of Pathobiology, University of Guelph, Guelph, ON N1G 2W1 Canada; Department of Population Medicine, University of Guelph, Guelph, ON N1G 2W1 Canada; Department of Clinical Studies, University of Guelph, Guelph, ON N1G 2W1 Canada

## Abstract

Annexins A1 and A2 are proteins known to function in the stress response, dampening inflammatory responses and mediating fibrinolysis. We found, in healthy cattle recently arrived to a feedlot, that lower levels of these proteins correlated with later development of pneumonia. Here we determine the localization of annexin A1 and A2 proteins in the respiratory tract and in leukocytes, in healthy calves and those with *Mannheimia haemolytica* pneumonia. In healthy calves, immunohistochemistry revealed cytoplasmic expression of annexin A1 in the surface epithelium of large airways, tracheobronchial glands and goblet cells, to a lesser degree in small airways, but not in alveolar epithelium. Immunocytochemistry labeled annexin A1 in the cytoplasm of neutrophils from blood and bronchoalveolar lavage fluid, while minimal surface expression was detected by flow cytometry in monocytes, macrophages and lymphocytes. Annexin A2 expression was detected in surface epithelium of small airways, some mucosal lymphocytes, and endothelium, with weak expression in large airways, tracheobronchial glands and alveolar septa. For both proteins, the level of expression was similar in tissues collected five days after intrabronchial challenge with *M. haemolytica* compared to that from sham-inoculated calves. Annexins A1 and A2 were both detected in leukocytes around foci of coagulative necrosis, and in necrotic cells in the center of these foci, as well as in areas outlined above. Thus, annexins A1 and A2 are proteins produced by airway epithelial cells that may prevent inflammation in the healthy lung and be relevant to development of pneumonia in stressed cattle.

**Electronic supplementary material:**

The online version of this article (doi:10.1186/s13567-014-0134-3) contains supplementary material, which is available to authorized users.

## Introduction

Annexins A1 and A2 are abundant proteins in bronchoalveolar lavage (BALF) of healthy calves, and lower levels in healthy at-risk calves were recently found to correlate with later development of bovine respiratory disease [1]. Annexin A1 and A2 are thought to quell inflammatory responses, and may thus promote resolution of inflammation and limit its injurious effects. Specifically, annexin A1 inhibits phospholipase A2 and eicosanoid synthesis, dampens neutrophil inflammatory responses, promotes neutrophil apoptosis, and stimulates interleukin-10 secretion from macrophages [2-7]. Annexin A2 activates plasminogen and thereby leads to fibrinolysis, enhances macrophage-mediated phagocytosis of apoptotic cells, and promotes airway epithelial cell repair [8-10]. Annexin A1 and A2 expression levels vary in different tissues [11]. An in vitro study of bovine tracheal epithelial cell cultures showed that annexin A1 was mostly expressed in differentiated cells and annexin A2 in undifferentiated cells, perhaps reflecting the anti-inflammatory and regenerative functions, respectively, of these proteins [12].

Annexin A1 levels increase with transportation stress [13], consistent with in vitro findings of increased annexin A1 and A2 expression after corticosteroid treatment of cultured bovine tracheal epithelial cells [12]. Recently, we found that as calves that were stressed by weaning and transportation arrived to a feedlot, those with higher levels of annexin A1 and A2 were less likely to later develop bacterial pneumonia [1]. Thus, the major objective of the present study was to determine the localization of annexins A1 and A2 in the respiratory tract of healthy calves, as well as to characterize differences that occur in inflamed lungs as a result of bacterial infection. This knowledge is necessary to understand how anti-inflammatory responses develop in the lung, and for development of methods to modulate these responses for prevention of bovine respiratory disease.

## Materials and methods

### Animals and sample collection

Samples of normal respiratory tissues were collected from two 2-month-old healthy male Holstein calves within 3 h of euthanasia. Samples of nasal tissue, trachea, bronchi, and lung containing alveoli and bronchioles were fixed in formalin overnight and processed routinely.

Further samples were collected from Holstein bull calves that were experimentally infected with *M. haemolytica* and from sham-inoculated control calves. Procedures were approved by the University of Guelph Animal Care Committee (AUP #12R055). Six Holstein bull calves, 102–139 days of age, were randomly assigned to two groups. *M. haemolytica*, isolated originally from a calf with pneumonia, was grown to log phase and diluted in PBS to optical density of 0.74 (shown to contain 2.8 × 10^8^ CFU/mL as determined by follow-up colony counts). Three calves were sedated with xylazine, and inoculated with 25 mL of inoculum (total dose of 7 × 10^9^ colony-forming units of *M. haemolytica* in phosphate-buffered saline) using a bronchoscope passed to the tracheal bifurcation. The 3 other calves, serving as uninfected controls, were similarly inoculated with 25 mL PBS. All infected calves developed moderately severe depression, reduced appetite and fever, as well as ultrasonographic evidence of consolidation within 2 hours of challenge that was maximal at 24 h, and elevated serum haptoglobin levels that peaked at 60 – 72 h after experimental challenge (data not shown). Calves were euthanized after 5 days. Tracheal, bronchial and lung tissues were fixed overnight in formalin and processed routinely.

Blood and BALF cells were collected from two healthy 2-3 month old Holstein male calves for immunocytochemistry. For flow cytometry, peripheral blood was collected from eight healthy adult Holstein cows after their first parturition.

### Immunohistochemistry and immunocytochemistry

For immunohistochemistry (IHC), tissue sections were prepared routinely, deparaffinized and rehydrated. Heat-induced epitope retrieval was performed using a pressure cooker with sodium citrate buffer (pH 6.0) at a pressure of 18 pounds per square inch to achieve a temperature of approximately 120 °C at full pressure (Dako North America, California, USA). Cooled slides were washed and treated with endogenous enzyme blocker and serum-free protein blocker (S2003 and X0909, Dako). The slides were incubated overnight with the primary antibody in a humidified chamber at 4 °C, using either rabbit polyclonal anti-human annexin A1 primary antibody (2 μg/150 μL; H00000301-D01P, Novus Biologicals, Oakville, ON, Canada) or goat polyclonal anti-human annexin A2 primary antibody (1 μg/150 μL; NB 100-881, Novus Biologicals). Slides were then washed and incubated with secondary antibody for 30 min, using peroxidase-based EnVision™+Dual Link Kit (#K406511-2, Dako Cytomation, Carpinteria, California, USA) for annexin A1, or HRP-conjugated rabbit anti-goat immunoglobulin (1:2000, Dako Cytomation) for annexin A2. NovaRED (Vector Laboratories, Burlingame, California, USA) was used as chromogen with Harris hematoxylin counterstain.

For immunocytochemistry (ICC), leukocytes were separated from whole blood by hypotonic lysis and cytocentrifuge preparations were made on charged slides. Preparations of BALF leukocytes were made in the same way. The cell preparations were fixed in acetone, and annexin A1 was detected as described above.

As negative controls for IHC and ICC, rabbit polyclonal anti-Toxoplasma antibody (2 μg/150 μL) was used in place of primary antibody for annexin A1, and goat polyclonal anti-influenza antibody (1 μg/150 μL) was similarly used as the negative control for annexin A2. As a further negative control, annexin A1 antibody was pre-incubated with bovine native annexin A1 protein (MBS318252, MybioSource, San Diego, CA, USA) for 1 h, using 1 μg antibody per 2.5 μg antigen for IHC, or 1 μg antibody per 1.5 μg antigen for 1 h for ICC. The IHC and ICC procedures were completed as above.

Immunolabeled slides were randomized and masked so that the assessment was done without knowledge of animal identity or treatment group. A semi-quantitative system was used to score each component of the respiratory system for intensity of immunolabeling (0, no staining; 1, equivocal; 2, minimal; 3, moderate; 4, abundant).

### Flow cytometry

Leukocytes were separated from red blood cells as above then incubated with rabbit polyclonal anti-human annexin A1 antibody (H00000301-D01P, Novus Biologicals) at a concentration of 0.1 mg/mL, for 2 h at 4 °C. Cells were washed once then incubated for 15 min with sheep anti-rabbit immunoglobulin antibody conjugated to R-phycoerythrin (1:5 dilution, STAR35A, AbD Serotec, Raleigh, USA) at 4 °C for 15 min. After washing, the cells were evaluated using a BD FACScanTM (BD Biosciences, USA) with acquisition of 300 000 events and analyzed using FlowJo software (version 7.6, Treestar Inc, Standord, CA) [14].

### Statistical analysis

The median fluorescence intensity (MFI) of leukocytes (neutrophils, lymphocytes and monocytes) was compared using Student’s *t* test (GraphPad, Prism 6) and considered significant at *P* < 0.05.

## Results

### Annexin A1 and A2 expression in respiratory tissues of healthy calves

**Figure 1:**
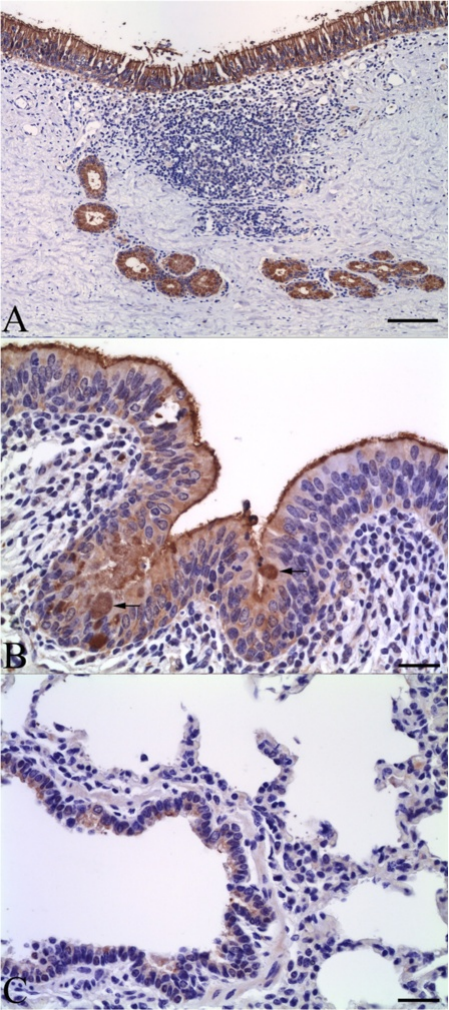
**Immunohistochemistry for annexin A1 in normal tissues. (A)** Trachea, with intense labeling of surface epithelium and mucosal glands. Bar = 100 μm. **(B)** Bronchus, with labeling of surface epithelium including cilia and goblet cells (arrows). Bar = 70 μm. **(C)** Weaker labeling of bronchiolar epithelium. Bar = 50 μm.

Immunohistochemistry findings were similar in sections of respiratory tissues from the two healthy calves (see Additional file 1). Annexin A1 immunolabeling was detected in the surface epithelium of the nasal cavity, trachea, bronchi and bronchioles, in both the apical and basal areas of the cytoplasm. The signal intensity was highest in the epithelium lining the nasal cavity, trachea (Figure 1A) and large bronchi (Figure 1B), lower in small bronchi and large bronchioles (Figure 1C), and infrequent in the terminal bronchioles. Labeling was also detected in cilia, goblet cells, and glandular and ductular epithelium of tracheobronchial glands (Figures 1A and B). Alveolar macrophages and some tracheobronchial mucosal lymphocytes were positively labeled, but cells in the alveolar septa were not labeled (Additional file 1F). Immunolabeling was noted in the airway smooth muscle and nerve terminals, but no labeling of connective tissue was observed in the bronchial adventitia or the lamina propria. Pre-incubation of annexin A1 antibody with annexin native protein abrogated the signal intensity (Additional file 1E). Labeling was not observed in sections treated with the control antibody in place of the primary antibody (Additional file 1B).

**Figure 2:**
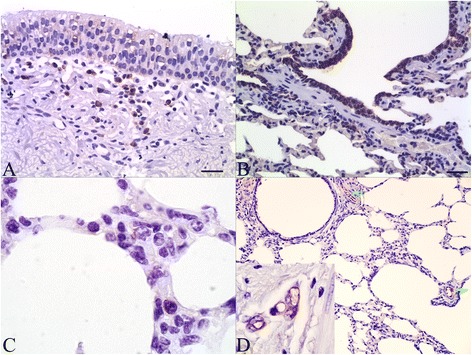
**Immunohistochemistry for annexin A2 in normal tissues. (A)** Trachea, with strong labeling of leukocytes but weak labeling of epithelium. Bar = 30 μm. **(B)** Bronchiole, with prominent labeling of epithelial cells. Bar = 30 μm **(C)** Labeling of individual cells in an alveolar septum consistent with macrophages or endothelial cells. **(D)** Absence of labeling of alveolar septa. Blood vessels (arrows) and bronchiolar epithelium are labeled. Bar = 50 μm. Inset: Intense labeling of endothelial cells in the lamina propria of a bronchus.

Annexin A2 immunolabeling was detected in epithelial cells lining the trachea, bronchi, bronchioles and alveoli, in both apical and basal areas of the cytoplasm (Additional file 2 and Additional file 3). The intensity of the signal was weak in the surface epithelium of the trachea and bronchi (Figure 2A). Bronchioles in most areas had intense labeling that was greater than that seen in the large airways, but this was not uniform throughout the sections (Figures 2B and D). The tracheobronchial glands were weakly labeled, and goblet cells or cilia were not labeled (Additional file 2A and Additional file 3A). Large lymphocytes in the tracheobronchial lamina propria were positively labeled (Figure 2A). Although the alveolar epithelium appeared negative, individual cells (suggestive of macrophages and endothelial cells) in alveolar septa were labeled (Figure 2C). Intense immunolabeling was observed in the endothelium of blood vessels (Figure 2D) and in plasma. Weak immunolabeling was noted in the airway smooth muscle and nerve terminals, and no labeling was observed in connective tissue of the bronchial adventitia or lamina propria. Labeling was not observed in sections treated with the negative control antibody in place of the primary antibody (Additional file 2C).

### Annexin A1 and A2 expression in lung following *M. haemolytica* challenge

**Figure 3:**
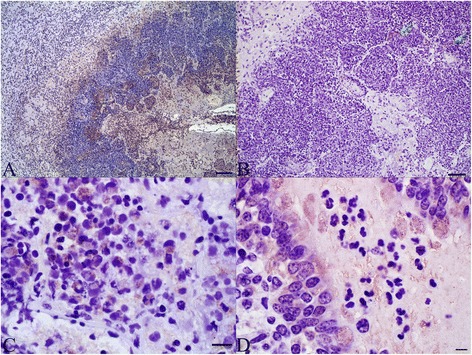
**Immunohistochemistry for annexins A1 and A2 in calves challenged with** ***M. haemolytica*** **. (A)** Annexin A1 expression in leukocytes within a focal area of coagulation necrosis (lower right) and in the band of leukocytes surrounding it. Bar = 100 μm. **(B)** Scant annexin A2 expression within necrotic cells, probably leukocytes (arrows), surrounding the focal area of necrosis. Bar = 50 μm. **(C)** Labeling of annexin A1 in necrotic leukocytes bordering an area of necrosis. Bar = 25 μm. **(D)** In an inflamed bronchiole, there is annexin A1 labeling of goblet cells including those that have been shed from the epithelium, but exudate neutrophils are unlabeled. Bar = 15 μm.

Immunohistochemical labeling of annexins A1 and A2 were compared in calves challenged with *M. haemolytica* and sham-challenged animals (Additional file 4 and Additional file 5). The histopathologic findings included foci of coagulative necrosis surrounded by a rim of necrotic leukocytes, the presence of fibrin and neutrophils within alveoli, and sloughed epithelial cells and infiltration of leukocytes into the lumen of airways. The necrotic foci and the leukocytes around the necrotic foci had patchy areas of immunolabeling for annexin A1 and to a minor extent for annexin A2 (Figures 3A and B). Annexin A1 labeling was intense within neutrophils at the center of the necrotic foci (Figure 3C). In contrast, neutrophils within bronchioles and alveoli were weakly and inconsistently labeled (Figure 3D). There was shedding of goblet cells from the bronchial epithelium and these goblet cells were intensely labeled for annexin A1 (Figure 3D). Subjectively, annexin A1 labeling of the epithelium of terminal bronchioles was more frequently detected in *M. haemolytica*- vs. sham-challenged animals, but this was not confirmed by the blinded objective evaluation (Additional file 6). Labeling for annexins A1 and A2 in the large airways was not significantly different between the calves challenged with *M. haemolytica* compared to the sham-challenged calves. Use of the negative control antibody did not result in labeling (Additional file 4C and E).

### Annexin A1 expression in blood and BALF leukocytes

**Figure 4:**
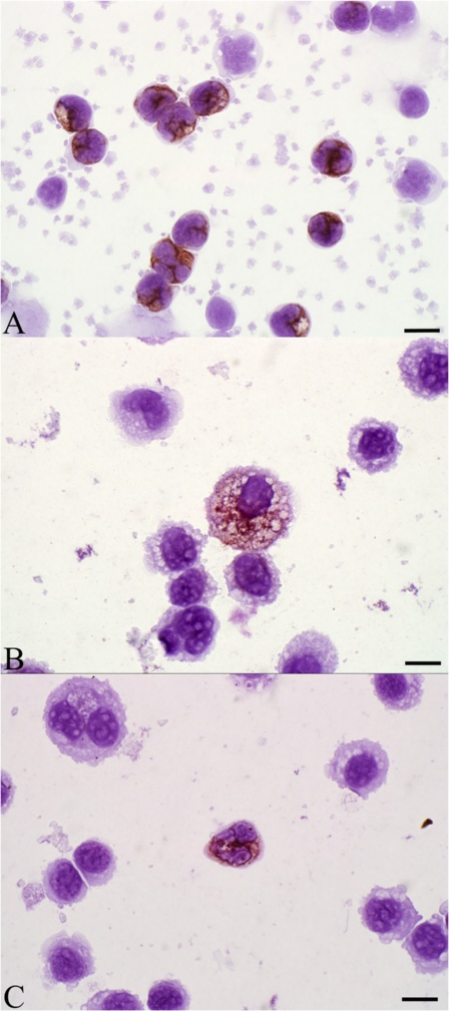
**Immunocytochemistry for annexin A1. (A)** Neutrophils in blood labeled for annexin A1; monocytes, lymphocytes and platelets are unlabeled. Bar = 10 μm. **(B)** Bronchoalveolar lavage fluid (BALF); Annexin A1 labeling in the cytoplasm of large foamy macrophages, but not in other macrophages. Bar = 10 μm. **(C)** BALF; neutrophils are rare in normal BALF but those present are labeled for annexin A1. Bar = 10 μm.

In normal blood leukocytes, immunocytochemistry showed positive labeling for annexin A1 in 100% of neutrophils (Figure 4A). The signal was detected in the cytoplasm, and cell membrane labeling was not observed. Lymphocytes, monocytes and platelets were not labeled. Adsorption of the primary antibody with annexin A1 protein abrogated the immunolabeling of the neutrophils (Additional file 7C).

In BALF, annexin A1 was not detected in most macrophages, nor in any lymphocytes (Additional file 7). However, annexin A1 signal was detected in low numbers of large BAL macrophages with abundant vacuolated cytoplasm (Figure 4B) and in large macrophages with features suggestive of apoptosis. Neutrophils were rare in cytocentrifuge preparations from the BALF of healthy calves, but those neutrophils present had intense labeling for annexin A1 (Figure 4C).

**Figure 5:**
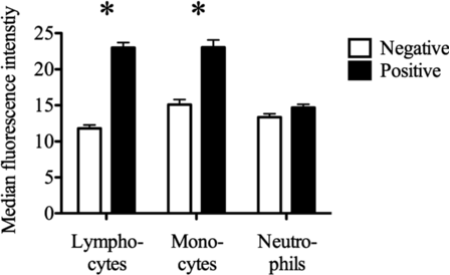
**Flow cytometric detection of annexin A1 on the surface of blood leukocytes.** Positive: antibody against annexin A1; negative: omission of the primary antibody. Median ± SEM from 8 animals. The MFI was significantly different from the negative control for lymphocytes (*P* < 0.0001) and monocytes (*P* = 0.0003) but not for neutrophils (*P* = 0.366).

Blood leukocytes were analyzed by flow cytometry for cell-surface expression of annexin A1 (Figure 5, Additional file 8). Among the leukocytes, 26% of lymphocytes and 18% of monocytes had median fluorescence intensity (MFI) above that seen in the negative control. The difference in MFI was significant in lymphocytes and monocytes (*P* < 0.0001 and *P* = 0.0003, Student’s *t*-test). In contrast, neutrophils had little or no shift in the MFI (*P* = 0.366) indicating lack of surface expression of annexin A1.

## Discussion

We investigated the distribution of annexin A1 and A2 protein within the respiratory tract of healthy cattle, and compared this to the levels found after experimental infection with *M. haemolytica*. In healthy calves, immunolabeling for annexins A1 and A2 was detected throughout the respiratory tract, especially in the airway surface epithelium and submucosal glands. Subtle differences in distribution and intensity of expression were noted for these two proteins: annexin A1 labeling was greatest in the surface and glandular epithelium of large proximal airways, and was present in goblet cells but not alveoli or endothelium; whereas annexin A2 labeling was weak in both surface epithelium of proximal and distal small airways, and was more intense in leukocytes and in endothelial cells. We found similar levels of both annexins A1 and A2 in undifferentiated club cells compared to differentiated ciliated epithelial cells and goblet cells of the trachea and bronchi, in contrast to previous work that was based on undifferentiated and differentiated (secretory) cells cultured in vitro [12]. The findings imply that the annexin A1 and A2 protein detected in bronchoalveolar lavage fluid of healthy calves is secreted by airway epithelial cells, since neutrophils are rare in the lumen of normal airways. Thus, annexin A1 and A2 are among the products secreted by airway epithelial cells that maintain an anti-inflammatory state in the healthy respiratory tract, and regulation of these responses may influence clinical outcome in cattle experiencing risk factors for pneumonia.

Comparison of calves experimentally infected with *M. haemolytica* and sham-inoculated calves showed little or no difference in the localization or intensity of immunolabeling for annexin A1 and A2. This argues against but does not eliminate the possibility that annexin gene expression is induced by inflammation, since steady state levels could be achieved if protein synthesis and secretion were both upregulated. Measuring annexin A1 levels in BALF of calves with pneumonia would address this possibility. However, the calves infected with *M. haemolytica* showed intense labeling of annexin A1 in leukocytes (mainly degenerating neutrophils) and BALF cells undergoing apoptosis. Conversely, exudate neutrophils within the lumen of airways were not labeled, perhaps as a result of activation-induced secretion of this protein. These findings suggest that during inflammation, annexin A1 released from leukocytes may contribute to the amount of this protein present in the inflamed tissue. The findings show no change in annexin A1 immunolabeling in lung parenchymal cells during pneumonia, but suggest increased levels of annexin A1 protein in the lung as a result of leukocyte infiltration. These data are consistent with prior detection of annexin A1 in the BALF of normal calves and those with experimentally induced bacterial and viral pneumonia [15,16].

The immunocytochemistry and flow cytometry results showed annexin A1 immunolabeling in the cytoplasm of normal blood and BALF neutrophils, but not on the cell surface. These findings concur with findings in human blood leukocytes [17], where annexin A1 was abundant in the cytoplasm of neutrophils, monocytes and natural killer cells but surface expression was not detected, and both surface and cytoplasmic expression was low in lymphocytes.

The flow cytometric analysis showed that, in the resting state, only a small amount of annexin A1 was present on the surface of lymphocytes and monocytes, and neutrophils had no detectable cell-surface expression of annexin A1. In contrast, studies of human leukocytes have shown low but detectable surface expression of annexin A1 on neutrophils [18]. The detection of annexin A1 in the cytoplasm of a subset of BAL macrophages does not necessarily indicate synthesis by these cells. Instead, annexin A1 immunolabeling in large foamy macrophages may have resulted from pinocytosis of airway secretions or phagocytosis of epithelial cells or neutrophils that contained this protein.

Annexin A1 has several known anti-inflammatory functions: it inhibits transendothelial migration and activation of neutrophils, enhances neutrophil apoptosis, inhibits activation of MAP kinase-mediated signal transduction, downregulates synthesis of inflammatory eicosanoids, and enhances secretion of the anti-inflammatory cytokine interleukin-10 from macrophages [2-7]. Similarly, the functions of annexin A2 include activation of plasminogen to initiate fibrinolysis, enhancing macrophage-mediated phagocytosis of apoptotic leukocytes, and enhancing repair of airway epithelial cells [8-10]. The finding that annexins A1 and A2 are mainly derived from airway epithelial cells in healthy individuals furthers understanding of how these cells dampen inflammation in the healthy airway and lung. Thus, although airway epithelial cells are capable of producing pro-inflammatory mediators and initiating airway host defenses, the resting airway epithelium appears to maintain homeostasis and the non-inflamed state of the healthy lung. Despite constant inhalation of organic dusts and bacteria, inflammation in the lung appears to be limited by secretion of annexins A1 and A2, club cell secretory protein, odorant binding protein, chitinase-like proteins, surfactant proteins A and D, lipoxins and resolvins, as well as epithelial cell surface expression of integrins and CD200 [19-21]. This concept suggests that dysregulation of these effects in airway epithelial cells could lead to tissue damage from the ensuing inflammatory response. This may explain the prior findings that calves with higher levels of annexins A1 and A2 in BALF had reduced prevalence of clinically apparent pneumonia.

In conclusion, annexin A1 immunolabeling was found to be most prominent in the surface epithelium, goblet cells and submucosal glands of the large proximal airways, and was also detected in the epithelium of distal airways, blood and BALF neutrophils, and large foamy macrophages in BALF. Annexin A2 immunolabeling was most abundant in the epithelial cells of the distal airways and in endothelial cells, with labeling also detected in alveoli and proximal airways, as well as in leukocytes and endothelial cells. The pattern and intensity of expression in the lung parenchyma was similar in calves with experimentally induced *M. haemolytica* pneumonia as in sham-inoculated calves, for both annexin A1 and A2. These findings imply that annexin A1 in the BALF of healthy calves mainly originates from the airway epithelium, whereas annexin A2 in BALF arises from airway epithelium and mucosal leukocytes. In BALF from inflamed lung, annexins A1 and A2 may also originate from leukocytes.

## Additional files

Additional file 1:
**Immunohistochemistry for annexin A1 in normal tissues.** (A) Trachea, with intense labeling of surface epithelium and tracheal glands. (B) Trachea; absence of labeling with the control antibody against *Toxoplasma*. (C) Bronchus, with labeling of surface epithelium including cilia and goblet cells (arrows). Inset: negative control (pre-incubation of antibody with antigen). (D) Weaker labeling of bronchiolar epithelium. (E) Bronchiole, negative control (prior adsorption of primary antibody with annexin protein) with partially abrogated labeling. (F) Absence of labeling of alveolar septa; minor labeling of bronchiolar epithelium. Additional file 2:
**Immunohistochemistry for annexin A2 in normal tissues.** (A) Trachea, with labeling of surface epithelium, mucosal glands, and lymphocytes in the lamina propria. Goblet cells or cilia of the respiratory epithelium were not labeled (arrow). (B) Bronchus, similar labeling as in the trachea. (C) Bronchus. Absence of labeling in the negative control using antibody against influenza virus. (D) Trachea, with strong labeling of leukocytes but weak labeling of epithelium. (E) Bronchus, similar labeling as in the trachea. (F) Bronchiole, with prominent labeling of the epithelium. Additional file 3:
**Immunohistochemistry for annexin A2 in normal tissues.** (A) Trachea, with labeling of tracheal glands. (B) Absence of labeling of alveolar septa. Blood vessels (arrows) and bronchiolar epithelium is labeled. (C, D) Labeling of individual cells in the alveolar septa consistent with macrophages or endothelium. (E) Intense labeling of endothelial cells in the lamina propria of a bronchus. Additional file 4:
**Focal coagulation necrosis and inflammation in the lung of a calf challenged with** ***M. haemolytica.*** (A) Focal necrosis (lower right) separated from more normal lung (upper left) by a dense band of necrotic leukocytes. Hematoxylin and eosin. (B) Annexin A1 expression in leukocytes in the necrotic lesion and in the band of leukocytes surrounding it. (C) Negative control for annexin A1 immunostain, using a primary antibody against Toxoplasma. (D) Scant annexin A2 expression in exudate surrounding the focal area of necrosis. (E) Negative control for annexin A2 immunostain, using a primary antibody against influenza virus. Additional file 5:
**Immunohistochemistry for annexin A1 and A2 in the lung of a calf challenged with** ***M. haemolytica.*** (A) Strong labelling of annexin A1 in necrotic leukocytes bordering an area of necrosis. (B) Weak labelling of annexin A2 in necrotic leukocytes bordering an area of necrosis. (C) Inflamed bronchiole, with moderate labeling for annexin A1. (D) Inflamed bronchiole, with weak labeling for annexin A2. (E) Inflamed bronchiole. There is annexin A1 labeling of goblet cells including those that have been shed from the epithelium, but exudate neutrophils are unlabeled. (F) Inflamed bronchiole. Annexin A2 labeling of bronchiolar exudate, but not of goblet cells or neutrophils. Additional file 6:
**Semi-quantitative scoring of immunohistochemical labeling of annexins A1 and A2 in the respiratory tract of six calves.** Three calves had been infected with *Mannheimia haemolytica* by intrabronchial challenge, and three were unchallenged controls. Additional file 7:
**Immunocytochemistry for annexin A1 in normal blood leukocytes and normal bronchoalveolar lavage fluid (BALF).** (A) Blood smear, Wright’s stain. (B) Neutrophils in blood labeled for annexin A1; monocytes and lymphocytes are unlabeled. (C) Negative control, using an antibody against Toxoplasma. (D) Wright’s stain of BALF leukocytes, showing macrophages and a single neutrophil. (E) BALF; Annexin A1 labeling in the cytoplasm of large foamy macrophages, but not in other macrophages. (F) BALF; neutrophils are rare in normal BALF but those present are labeled for annexin A1. Additional file 8:
**Flow cytometry for surface expression of annexin A1 in normal bovine blood leukocytes.** (A) Scatterplot, indicating forward- and side-scatter characteristics of neutrophils, lymphocytes and monocytes. (B, C, D) Histograms of fluorescence (annexin A1) vs. cell count for cells gated as neutrophils (B), lymphocytes (C) and monocytes (D). Blue: labeled with antibody against annexin A1; red: negative control (primary antibody omitted). (E) Flow cytometric detection of annexin A1 on the surface of blood leukocytes. Positive: antibody against annexin A1; negative: omission of the primary antibody. Median ± SEM from 8 animals. The MFI was significantly different from the negative control for lymphocytes (*P* < 0.0001) and monocytes (*P* = 0.0003) but not for neutrophils (*P* = 0.366).

## Abbreviations

ICC: Immunocytochemistry
IHC: Immunohistochemistry
MFI: Median fluorescence intensity
