# Supplementary figures and images for: Localization of annexins A1 and A2 in the respiratory tract of healthy calves and those experimentally infected with Mannheimia haemolytica
Source: Vet Res. 2015 Feb 14;46:6. doi: 10.1186/s13567-014-0134-3 (PMC4327810; doi:10.1186/s13567-014-0134-3)

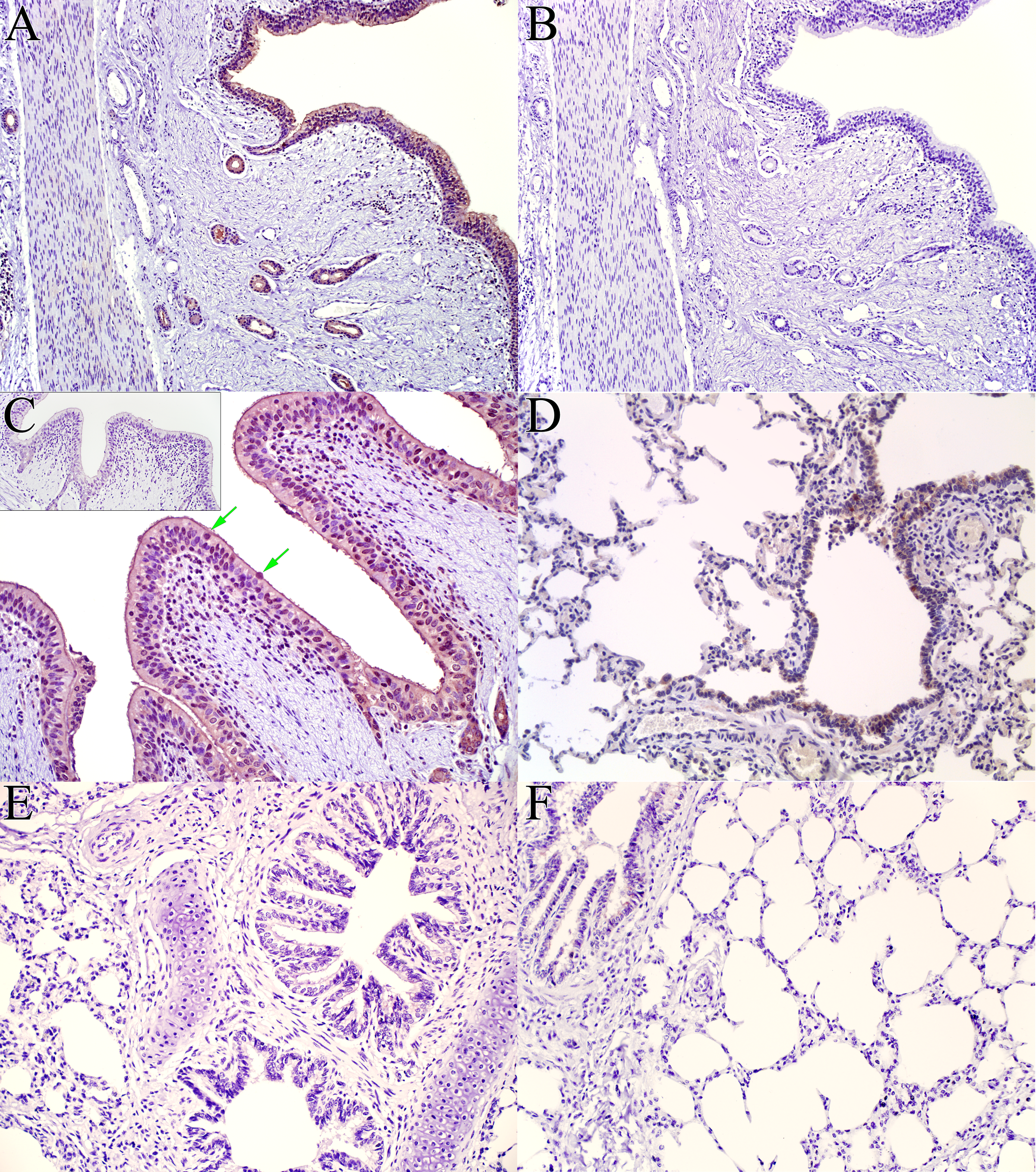

Supplement: Additional file 1: — Immunohistochemistry for annexin A1 in normal tissues. (A) Trachea, with intense labeling of surface epithelium and tracheal glands. (B) Trachea; absence of labeling with the control antibody against Toxoplasma. (C) Bronchus, with labeling of surface epithelium including cilia and goblet cells (arrows). Inset: negative control (pre-incubation of antibody with antigen). (D) Weaker labeling of bronchiolar epithelium. (E) Bronchiole, negative control (prior adsorption of primary antibody with annexin protein) with partially abrogated labeling. (F) Absence of labeling of alveolar septa; minor labeling of bronchiolar epithelium. [file 13567_2014_134_MOESM1_ESM.png]

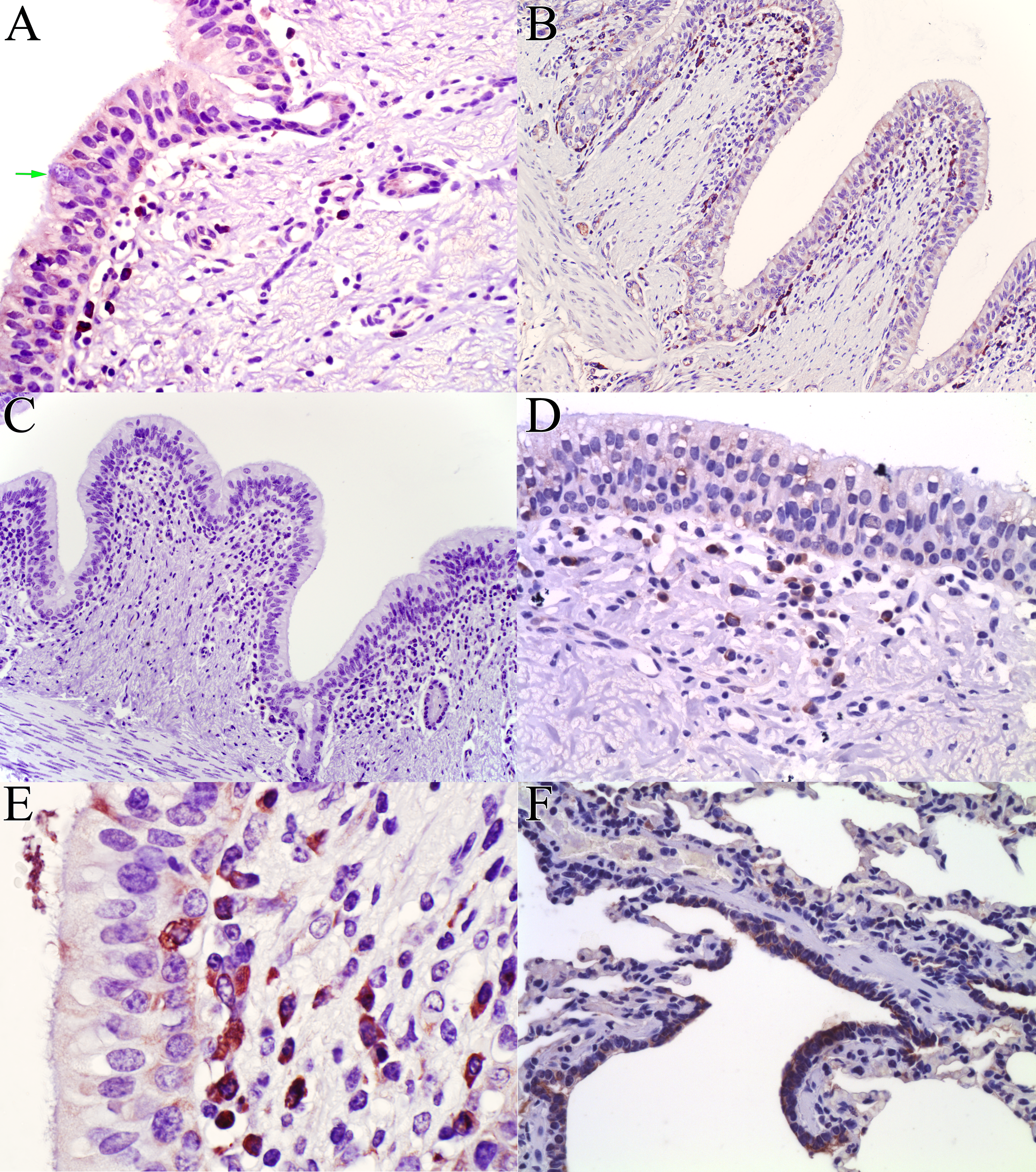

Supplement: Additional file 2: — Immunohistochemistry for annexin A2 in normal tissues. (A) Trachea, with labeling of surface epithelium, mucosal glands, and lymphocytes in the lamina propria. Goblet cells or cilia of the respiratory epithelium were not labeled (arrow). (B) Bronchus, similar labeling as in the trachea. (C) Bronchus. Absence of labeling in the negative control using antibody against influenza virus. (D) Trachea, with strong labeling of leukocytes but weak labeling of epithelium. (E) Bronchus, similar labeling as in the trachea. (F) Bronchiole, with prominent labeling of the epithelium. [file 13567_2014_134_MOESM2_ESM.png]

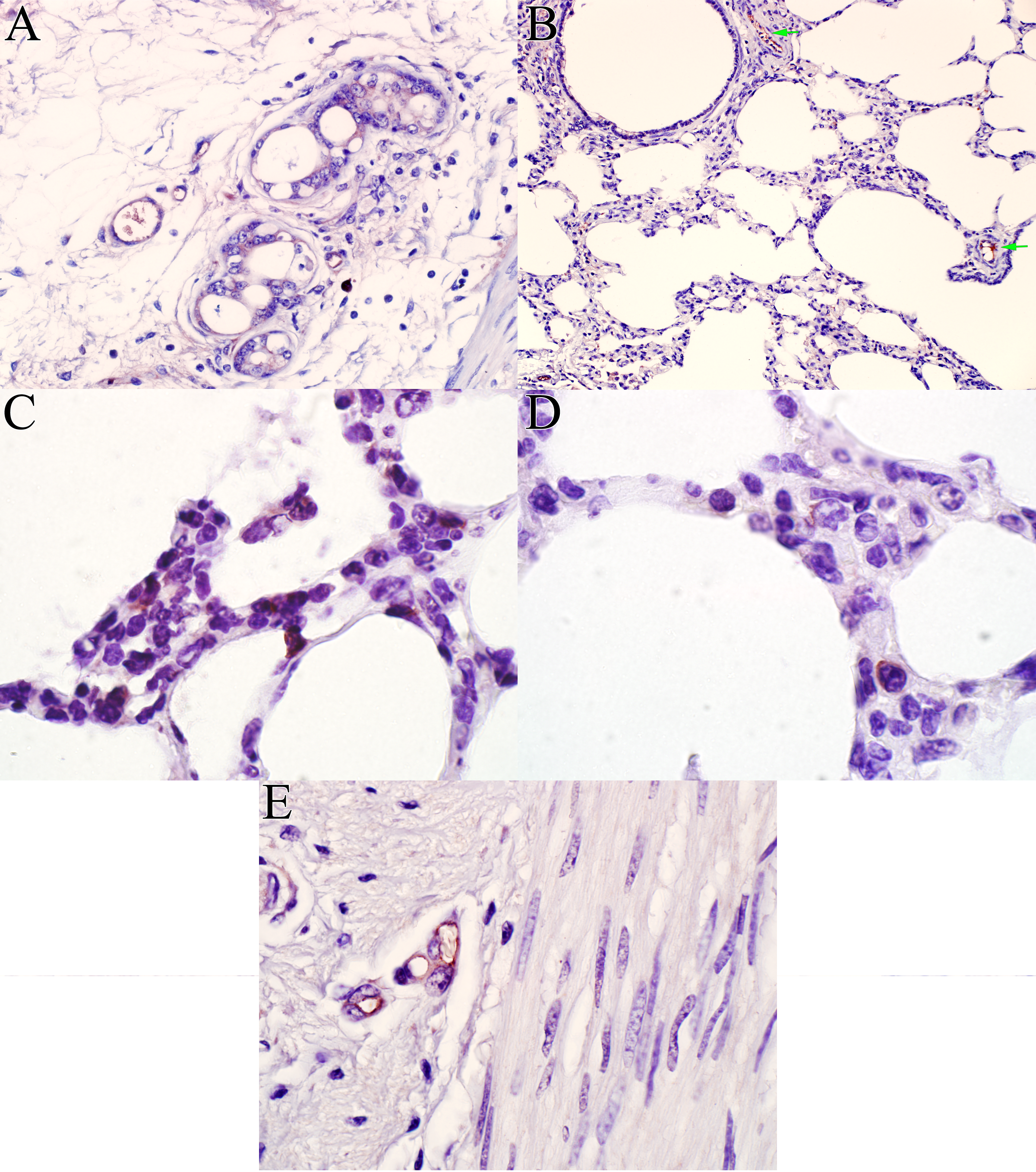

Supplement: Additional file 3: — Immunohistochemistry for annexin A2 in normal tissues. (A) Trachea, with labeling of tracheal glands. (B) Absence of labeling of alveolar septa. Blood vessels (arrows) and bronchiolar epithelium is labeled. (C, D) Labeling of individual cells in the alveolar septa consistent with macrophages or endothelium. (E) Intense labeling of endothelial cells in the lamina propria of a bronchus. [file 13567_2014_134_MOESM3_ESM.png]

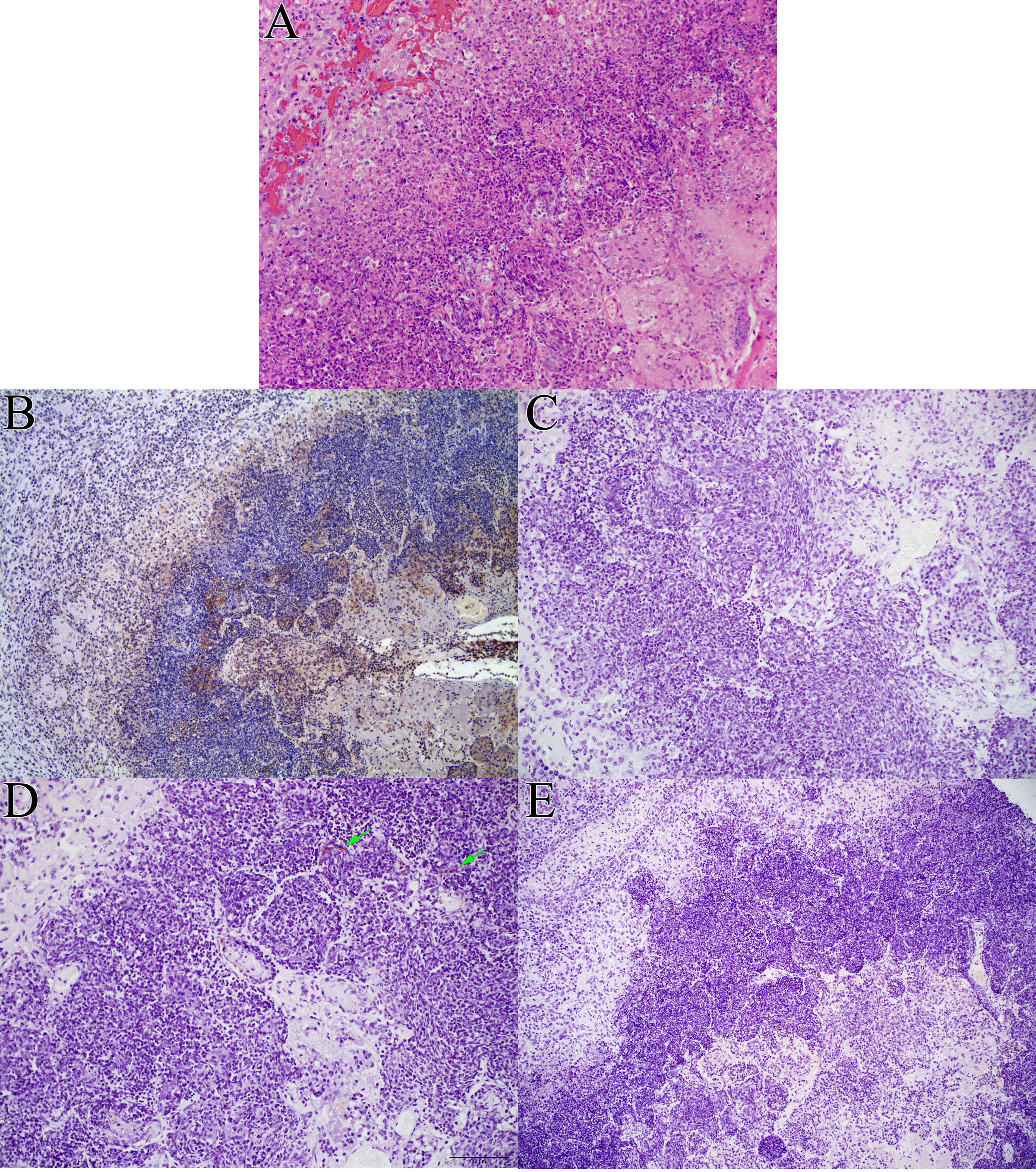

Supplement: Additional file 4: — Focal coagulation necrosis and inflammation in the lung of a calf challenged with M. haemolytica. (A) Focal necrosis (lower right) separated from more normal lung (upper left) by a dense band of necrotic leukocytes. Hematoxylin and eosin. (B) Annexin A1 expression in leukocytes in the necrotic lesion and in the band of leukocytes surrounding it. (C) Negative control for annexin A1 immunostain, using a primary antibody against Toxoplasma. (D) Scant annexin A2 expression in exudate surrounding the focal area of necrosis. (E) Negative control for annexin A2 immunostain, using a primary antibody against influenza virus. [file 13567_2014_134_MOESM4_ESM.tiff]

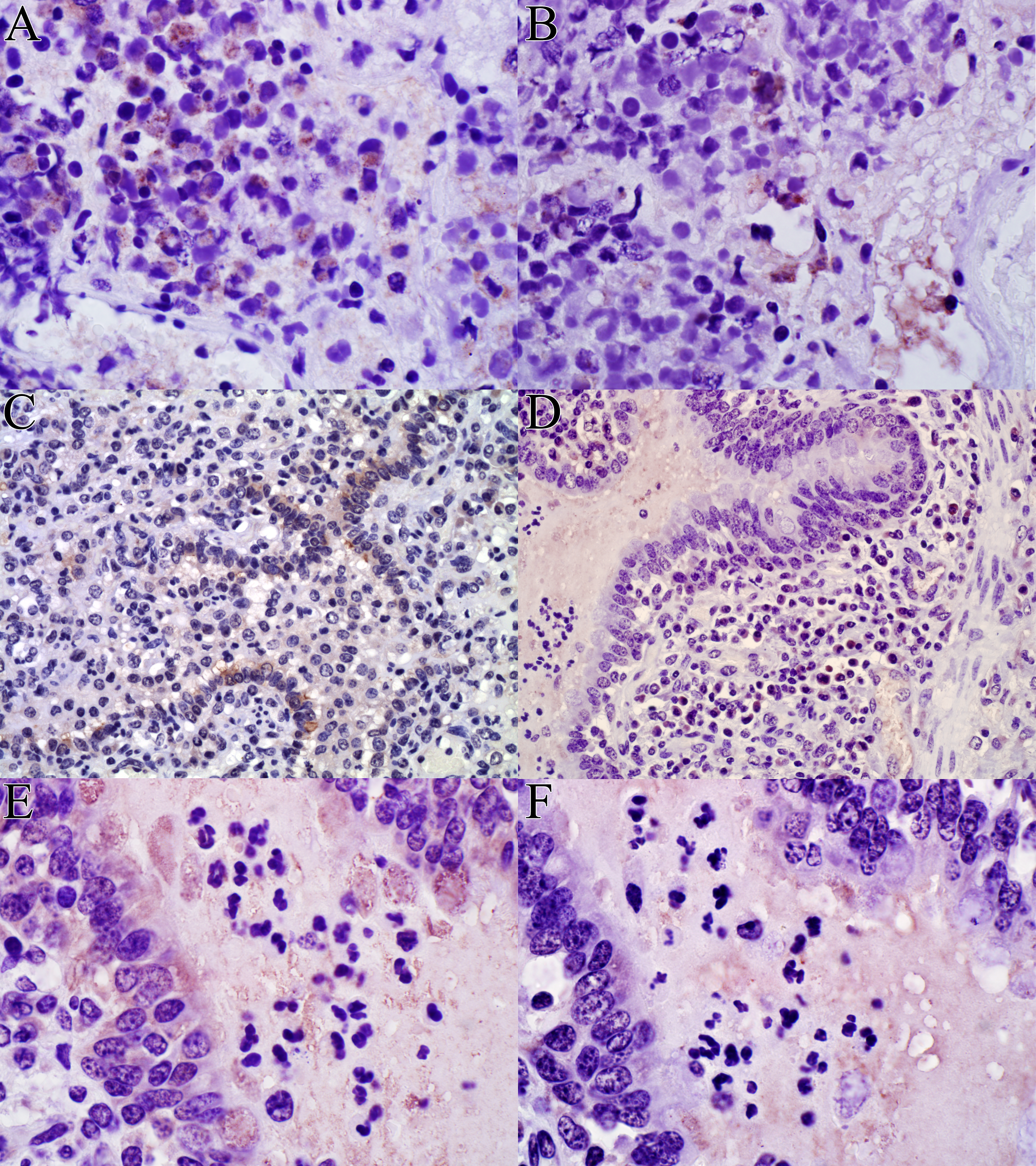

Supplement: Additional file 5: — Immunohistochemistry for annexin A1 and A2 in the lung of a calf challenged with M. haemolytica. (A) Strong labelling of annexin A1 in necrotic leukocytes bordering an area of necrosis. (B) Weak labelling of annexin A2 in necrotic leukocytes bordering an area of necrosis. (C) Inflamed bronchiole, with moderate labeling for annexin A1. (D) Inflamed bronchiole, with weak labeling for annexin A2. (E) Inflamed bronchiole. There is annexin A1 labeling of goblet cells including those that have been shed from the epithelium, but exudate neutrophils are unlabeled. (F) Inflamed bronchiole. Annexin A2 labeling of bronchiolar exudate, but not of goblet cells or neutrophils. [file 13567_2014_134_MOESM5_ESM.png]

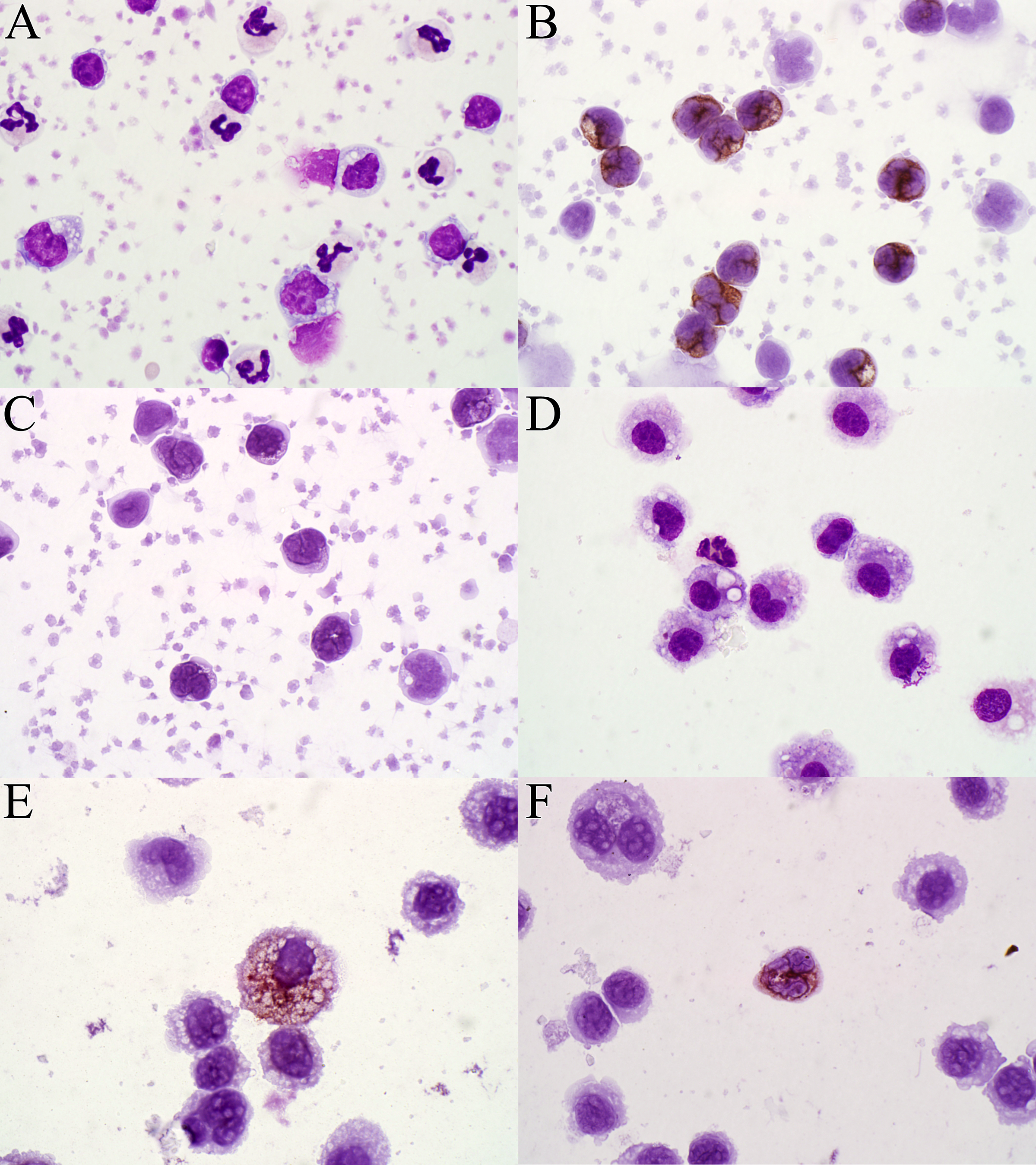

Supplement: Additional file 7: — Immunocytochemistry for annexin A1 in normal blood leukocytes and normal bronchoalveolar lavage fluid (BALF). (A) Blood smear, Wright’s stain. (B) Neutrophils in blood labeled for annexin A1; monocytes and lymphocytes are unlabeled. (C) Negative control, using an antibody against Toxoplasma. (D) Wright’s stain of BALF leukocytes, showing macrophages and a single neutrophil. (E) BALF; Annexin A1 labeling in the cytoplasm of large foamy macrophages, but not in other macrophages. (F) BALF; neutrophils are rare in normal BALF but those present are labeled for annexin A1. [file 13567_2014_134_MOESM7_ESM.png]

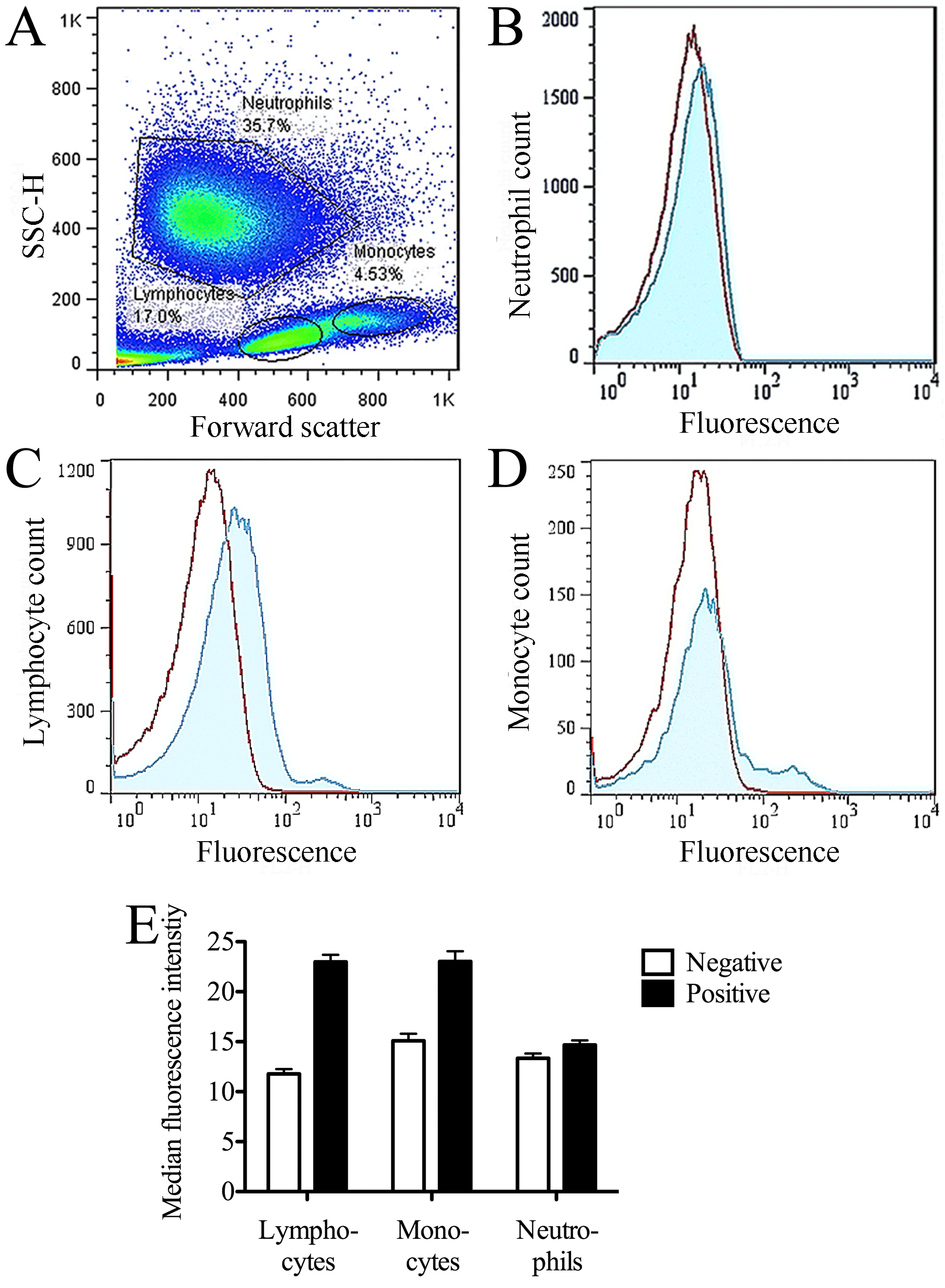

Supplement: Additional file 8: — Flow cytometry for surface expression of annexin A1 in normal bovine blood leukocytes. (A) Scatterplot, indicating forward- and side-scatter characteristics of neutrophils, lymphocytes and monocytes. (B, C, D) Histograms of fluorescence (annexin A1) vs. cell count for cells gated as neutrophils (B), lymphocytes (C) and monocytes (D). Blue: labeled with antibody against annexin A1; red: negative control (primary antibody omitted). (E) Flow cytometric detection of annexin A1 on the surface of blood leukocytes. Positive: antibody against annexin A1; negative: omission of the primary antibody. Median ± SEM from 8 animals. The MFI was significantly different from the negative control for lymphocytes (P < 0.0001) and monocytes (P = 0.0003) but not for neutrophils (P = 0.366). [file 13567_2014_134_MOESM8_ESM.png]
